# Simulation-based training in Leprosy: development and validation of a scenario for community health workers

**DOI:** 10.1590/0034-7167-2023-0114

**Published:** 2023-12-08

**Authors:** Raíssa Silva Souza, Juliana Almeida Menezes Moreira, Ana Angélica Lima Dias, Angélica da Conceição Oliveira Coelho, José Joaquim Penedos Amendoeira, Fernanda Moura Lanza

**Affiliations:** I Universidade Federal de São João del-Rei. Divinópolis, Minas Gerais, Brazil; II Universidade Federal de Juiz de Fora. Juiz de Fora, Minas Gerais, Brazil; III Instituto Politécnico de Santarém, Escola Superior de Saúde. Santarém, Portugal

**Keywords:** Simulation Training, Simulation, Leprosy, Community Health Workers, Primary Health Care, Entrenamiento Simulado, Simulación, Enfermedad de Hansen, Agentes Comunitarios de Salud, Atención Primaria de Salud, Treinamento por Simulação, Simulação, Hanseníase, Agentes Comunitários de Saúde, Atenção Primária à Saúde

## Abstract

**Objectives:**

To build and validate a clinical simulation scenario designed to instruct community health workers (CHWs) in active leprosy case detection.

**Methods:**

Methodological study involving the development of a simulated clinical scenario and content validation by experts. The Content Validity Index (CVI) was used to determine the level of agreement among the judging commitee, and a descriptive analysis of their recommendations was performed.

**Results:**

A simulated scenario with a simulated participant was developed — a simulation characterized by low complexity, moderate physical/environmental fidelity, moderate to high psychological fidelity, and high conceptual fidelity, lasting 50 minutes and capable of training up to 10 CHWs simultaneously. The scenario was validated by 14 experts, with a CVI exceeding 80% for all components.

**Conclusions:**

The validated clinical simulation possesses attributes that make it highly reproducible in various national health contexts, thereby contributing to the global “Towards Zero Leprosy” strategy.

## RESUMO

**Objetivos:**

Construir e validar cenário de simulação clínica para ensinar a agentes comunitários de saúde (ACS) ações de busca ativa da hanseníase.

**Métodos:**

Estudo metodológico de construção de cenário clínico simulado e de validação de conteúdo por especialistas. Utilizou-se o Índice de Validade de Conteúdo (IVC) para determinar o grau de concordância entre os juízes e a análise descritiva das recomendações.

**Resultados:**

Criou-se uma simulação cênica com participante simulado, de baixa complexidade, média fidelidade física/ambiental, média/alta fidelidade psicológica e alta fidelidade conceitual, cujo tempo de duração total é de 50 minutos, capaz de qualificar até dez ACS simultaneamente. Quatorze juízes validaram o cenário, obtendo-se IVC superior a 80% em todos os componentes.

**Conclusões:**

A simulação clínica validada tem atributos que a tornam altamente replicável em diferentes contextos de saúde nacionais, podendo, dessa forma, contribuir com a estratégia global “Rumo à zero hanseníase”.

## RESUMEN

**Objetivos:**

Construir y validar escenario de simulación clínica para enseñar a agentes comunitarios de salud (ACS) acciones de búsqueda activa de la lepra.

**Métodos:**

Estudio metodológico de construcción de escenario clínico simulado y de validez de contenido por especialistas. Utilizado el Índice de Validez de Contenido (IVC) para determinar el grado de concordancia entre los jueces y el análisis descriptivo de las recomendaciones.

**Resultados:**

Creado una simulación escénica con participante simulado, de baja complejidad, mediana fidelidad física/ambiental, mediana/alta fidelidad psicológica y alta fidelidad conceptual, cuyo tiempo de duración total fue de 50 minutos, capaz de calificar hasta diez ACS simultáneamente. Catorce jueces validaron el escenario, obteniéndose IVC superior a 80% en todos los componentes.

**Conclusiones:**

La simulación clínica validada tiene atributos que la transforman altamente replicable en diferentes contextos de salud nacionales, pudiendo, de esa manera, contribuir con la estrategia global “Rumbo al cero lepra”.

## INTRODUCTION

Leprosy (Hansen’s disease) persists as a public health issue in various countries, including Brazil, which continues to bear a high disease burden, ranking second in the world in new cases and first among the countries in the Americas^(1)^. Despite the decreasing trend in the annual detection rate, most new cases in the country are multibacillary, with visible impairments at diagnosis, including a substantial number of individuals under the age of 15 diagnosed with the disease. These epidemiological characteristics pose challenges to public health authorities^(2)^.

To attain the objectives outlined in the global strategy “Towards Zero Leprosy” for 2021-2030, the country must expedite its efforts to interrupt transmission and reach zero autochthonous cases^(1)^. This will require substantial efforts from Primary Health Care (PHC) services in developing and proposing more effective measures to combat the disease in Brazil^(3)^. Community health workers (CHWs), members of the Family Health Strategy (FHS), one of the PHC modalities in Brazil, are professionals who can actively contribute to leprosy control actions (LCAs)^(4, 5)^. This is because they have a direct connection to the community, which promotes the integration of health actions within the household context^(6)^.

Among the responsibilities of these professionals in interrupting the disease’s chain of transmission are active case-detecting activities carried out during home visits. During these visits, CHWs have the opportunity to promptly identify leprosy cases and raise awareness within the community about the disease^(4)^.To carry out these activities effectively, CHWs need to be trained and qualified^(5, 6)^.

However, it is noticeable that the pedagogical strategies employed in the training and qualification processes of CHWs primarily rely on the transmission of knowledge and information from official instructional documents, which must be uncritically assimilated by these individuals. In light of evidence^(^7-8^)^ demonstrating the insecurity of CHWs in carrying out LCAs, even after training, including those with a practical approach, it is presumed that the use of pedagogical approaches prioritizing ways of constructing knowledge based on the action-reflection-action triad^(9)^ has the potential to fill this gap.

One of these approaches, which has gained significant traction in the health sector in recent times, is the theory of experiential learning^(9)^, whose premise is based on the understanding that individuals can learn through conscious reflection on their experiences. Clinical simulation is a strategy that facilitates the application of this approach and is considered an international gold standard in health education, enhancing the quality of various caregiving processes^(10, 11)^.

For a simulation to occur, the development of a clinical scenario is necessary, which involves detailed planning of the stages of clinical simulation^(12)^. Such a scenario must create suitable conditions for the student to have realistic cognitive, psychomotor, and affective/relational experiences so that they can transfer the knowledge gained from clinical simulation to their professional practice^(12)^. However, it is essential to use previously structured and validated scenarios to ensure the quality of the instrument through integrity, reproducibility, and alignment with recommended standards for the construction of clinical simulation scenarios^(10, 13, 14)^.

The undertaking of this study is justified by the understanding that there is a knowledge gap related to the use of clinical simulation as a methodological tool in permanent health education (PHE) activities for CHWs and the need to strengthen the role of these professionals in carrying out LCAs to achieve the objectives outlined in the global strategy for 2021-2030^(1)^.

## OBJECTIVE

To build and validate a clinical simulation scenario for instructing community health workers in active leprosy case detection.

## METHODS

### Ethical aspects

The research protocol was approved by the Research Ethics Committee with Human Subjects of the Federal University of São João del-Rei. All participants provided consent by electronically signing the Informed Consent Form (ICF), which was made available in digital format.

### Study design, period, and location

The methodological study was carried out in two stages: the construction of a clinical simulation scenario and content validation by an expert judging committee in the relevant subject areas. The first stage occurred from March to August 2021, and the second stage was conducted in a virtual environment between September and October 2021. Being a methodological study, it is important to emphasize that none of the EQUATOR Network guidelines could be applied.

### Population, inclusion and exclusion criteria

The validation process with the expert committee occurred in a virtual environment using the Google Forms® platform. Only nurses were invited to participate in this stage, as according to the National Primary Care Policy (PNAB), they are the professionals responsible for planning, managing, supervising, and evaluating the actions carried out by CHWs within the PHC team^(4)^.

In this study, participants were intentionally selected, and the inclusion criteria were based on parameters adapted from Fehring^(15)^, which included having a postgraduate degree (either lato sensu or stricto sensu) and/or professional experience in the subject areas of the study (leprosy and/or clinical simulation). For professional experience, the following criteria were considered: (i) at least two years of clinical experience in the Family Health Strategy; and/or (ii) at least two years of experience in managing the Leprosy Control Program; and/or (iii) experience in university teaching, research, and/or extension activities in the study’s subject areas, with a scientific publication in the respective field within the last two years.

Experts were selected by reviewing the curricula available on the Lattes Platform of the Conselho Nacional de Desenvolvimento Científico e Tecnológico (CNPq, National Council for Scientific and Technological Development). Initially, 49 nurses with profiles matching the specified criteria (a minimum score of five points according to Fehring’s parameters^(15)^) were identified. They were invited to participate in the research through an invitation letter sent via email. In this email, potential participants were provided with a link to the Google Forms® platform, which contained the ICF, the expert characterization instrument, specific instructions on the procedures for the analysis and assessment of the clinical scenario’s content validity, the scenario itself, and a survey for suggesting additional experts for participation.

Of the 49 potential participants, 10 accepted the invitation, completed the instrument, and suggested six other nurses, of whom only four met Fehring’s parameters^(15)^ and agreed to participate.

A 15-day deadline was given for the completion of the instrument, and those who did not respond within this timeframe were contacted once again by email.

### Study protocol

A script was used for the construction of the simulated scenario^(14)^. It was formulated based on the theoretical framework of the National League for Nursing Jeffries Simulation Theory (NLN/JST)^(16)^, as well as the recommendations of the International Nursing Association for Clinical Simulation and Learning (INACSL)^(13)^. These frameworks are considered complementary, as NLN/JST establishes the conceptual components of the theoretical model, while INACSL defines the necessary items for the construction of clinical scenarios based on evidence in the field^(17)^. Regarding the theoretical framework for leprosy, the study relied on the official recommendations of the Ministry of Health (MH) concerning the CHW’s role in active case-finding actions for the disease^(4)^.

The NLN/JST outlines the conceptual components of clinical simulation. These include context; background; design; simulation experience; participant; facilitator and educational strategy; and outcomes^(16)^. They will be described in detail below.

“Context” encompasses the specifications of the assumptions underlying the simulation’s development and evaluation^(16)^. “Background” encompasses the learning objectives and expected outcomes in terms of competencies and communication skills, healthcare attention, decision-making, and leadership, which are essential for resolving the presented clinical situation^(16)^. “Design” specifies the elements of scenario preparation according to their complexity, degree of realism, and desired fidelity. Within it, the strategies for briefing and debriefing are detailed.

“Simulation experience” elucidates the attributes of the simulated experience in an experiential, interactive, collaborative, and learning-centered environment. “Facilitator” and “educational strategy” focus on the quality of interaction between the facilitator and the participant from the chosen theoretical perspective^(16)^. “Participant” explores the natural and innate attributes that can influence the simulation. Finally, “outcomes” refer to the assessment of participants’ comprehension of the information presented in the scenario and their simulation experience, including its impact on the participant, patient, and the system^(16)^.

The initial version of the clinical scenario, submitted for expert judging committee analysis and evaluation, contained the following elements: scenario description; learning objectives; expected outcomes; assessment method and strategy; modality; participant preparation and educational resources; estimated time of completion; prebriefing; briefing; participants and simulation team; materials, equipment, and simulators; characterization and scripts; scene progression; checklist; and debriefing.

For the scenario validation stage, the prepared material was subdivided into five sections within Google Forms®, which were analyzed according to all the criteria proposed by Pasquali^(18)^: (1) comprehensibility to the target population; (2) objectivity; (3) simplicity; (4) clarity; (5) relevance; (6) precision; (7) variety; (8) modality; (9) typicality; (10) credibility; (11) scope; and (12) balance. The assessment of each criterion was based on a Likert-type scale with four response alternatives: 1 = strongly disagree; 2 = disagree; 3 = agree; and 4 = strongly agree^(18)^. Space was provided at the end of each section for comments and suggestions.

### Analysis of results and statistics

The data were processed and analyzed using Microsoft Excel®, version 2019. To validate the five sections of the clinical scenario, the Content Validation Index (CVI) was calculated for each of Pasquali’s criteria. This is a measure of agreement among experts on the evaluated topics in the scenario, calculated by summing the responses graded as “3” and “4” on the Likert scale, divided by the total number of responses. Items with 80% or more agreement among experts were considered validated^(19)^.

## RESULTS

The clinical scenario “Actions for the prevention and control of leprosy: active search for dermatoneurological symptomatics” proposes the reproduction of a household using simple materials commonly found in PHC units to compose the scene. Thus, it can be classified as having moderate physical/environmental fidelity.

It also falls under high conceptual fidelity, as it utilizes updated recommendations from the MH and has been subjected to evaluation by experts in the thematic area. Additionally, it has moderate/high psychological fidelity, depending on how participants engage in maintaining the fictional contract.

Other fidelity aspects in the scenario include the detailed creation of *moulage* and the methods of training and preparing the simulated patient, who may even be a resident of the CHW’s own area of operation (if willing to be trained to participate in clinical simulation). The complete script for preparing the simulated patient, which contains aspects of personal life, current illness, health concerns, and lifestyle habits of the character to be portrayed, is integrated into the scenario to ensure standardized representation and the achievement of proposed objectives.

Therefore, it is a scenic simulation with a simulated participant aimed at training the CHW to actively seek for dermatoneurological symptomatic individuals in their operational territory. The learning objectives are: (i) to conduct health education activities on the signs and symptoms of leprosy; (ii) to eliminate misconceptions about leprosy, emphasizing local culture as a point of contact for building new knowledge about the disease; (iii) to identify dermatoneurological complaints during home visits, recognizing that they may be signs and symptoms of leprosy; (iv) to recognize the need to refer dermatoneurological symptomatic patients for evaluation at PHC units; and (v) to refer suspected cases of leprosy to PHC units for scheduling their dermatoneurological assessments. Regarding the achievement of learning objectives, it can be classified as having low complexity.

The estimated time for activity completion is 50 minutes, divided into prebriefing, briefing, scene execution, and debriefing moments. Furthermore, there is a planned and detailed participant preparation activity with the purpose of aligning their knowledge on the topic. This consists of a dialogue-based presentation with an estimated duration of 15 minutes.

The simulation team includes the facilitator and the simulated participant. Participants can be observers (up to nine) and a volunteer. The scene setting will be prepared to resemble a household to be visited by the volunteer participant (a CHW).

The scenario represents a case where a CHW (role of the volunteer participant) goes to a residence to conduct an active search for individuals with signs and symptoms suggestive of leprosy in their microarea of operation, as presented in Chart 1.

**Chart 1:** Briefing and scene progression of the clinical scenario “Actions for the prevention and control of leprosy: active search for dermatoneurological symptomatics”. Divinópolis, Minas Gerais, Brazil, 2021

**Briefing** 08:30 a.m. You, as a community health worker, have been instructed to actively seek out individuals with signs and symptoms suggestive of leprosy during your home visits in your microarea of operation. The first residence you will visit belongs to the Oliveira family, who are registered as number 48 in your microarea. This family has been residing in the microarea for three years, and according to the records, the Oliveira family consists of three members: Mr. Sebastião Oliveira, 34 years old, an informal construction worker; his wife, Rosana Oliveira, 36 years old, a homemaker; and their son, João Oliveira, 2 years old. You knock on their door to begin the visit.			
**Scene Progression**			
Duration	Expected actions of the volunteer participant	Simulated participant’s dialogue	Hints/Tips
0-2. minutes	Identify yourself to the simulated participant. Request the identification of the person (simulated participant) who is greeting you. Ask if the other members of the Oliveira family are at home. Inquire if the Oliveira family have any complaints or requests for the PHC unit.	Hello. Good morning! I am Sebastião. Rosana is not home right now. She and João went to visit my mother-in-law. We’re doing very well, and we’re not experiencing any issues at the moment.	Ask who knocked on your door. Ask the CHW for their name. Ask the CHW if it’s necessary for all family members to be at home to proceed with the visit. Inform the CHW that neither he nor any other family member has any complaints at the moment.

Chart 1 also displays the actions expected at the different stages of the scenario, suggestions for “dialogue”, hints, and tips that can be used by the simulation team.

Of the 14 participants in the clinical scenario’s content validation stage, eight (57%) were experts in the field of leprosy, and six (43%) in clinical simulation. They were predominantly female (*n* = 12; 86%), with an average age of 45 years, with more than 10 years of undergraduate education (*n* = 11; 79%); holding a doctoral degree (*n* = 10; 72%), and engaged in teaching, research, and extension activities (*n* = 10; 72%).

The result of the analysis and assessment of each section of the clinical scenario by the experts, according to the CVI obtained using Pasquali’s criteria^(18)^, is presented in Table 1.

**Table 1:** CVI results of the sections of the clinical scenario “Actions for the prevention and control of leprosy: active search for dermatoneurological symptomatics”. Divinópolis, Minas Gerais, Brazil, 2021

Pasquali’s assessment criteria	Clinical Scenario Sections				
	Section 1*	Section 2^†^	Section 3^‡^	Section 4^§^	Section 5^||^
Comprehensibility	100%	93%	100%	100%	100%
Objectivity	93%	100%	100%	100%	100%
Simplicity	100%	93%	100%	100%	100%
Clarity	100%	93%	100%	100%	100%
Relevance	100%	100%	100%	100%	100%
Precision	100%	93%	100%	100%	100%
Variety	100%	100%	100%	100%	100%
Modality	100%	100%	100%	100%	100%
Typicality	100%	100%	100%	100%	100%
Credibility	100%	100%	100%	100%	100%
Scope	100%	100%	100%	100%	100%
Balance	100%	100%	100%	100%	100%
**Result**	**Approved**	**Approved**	**Approved**	**Approved**	**Approved**

*Conceptual components of “Context” and “Background”; ^†^ Conceptual component of “Background”; ^‡^ Conceptual component of “Design” (prebriefing and briefing); ^§^ Conceptual component of “Design” (debriefing); ^||^ Conceptual components of “facilitator and educational strategy”, “simulated experience”, and “results”.

Considering that all criteria obtained a CVI greater than 80%, it can be recognized that the clinical scenario “Actions for the prevention and control of leprosy: active search for dermatoneurological symptomatics” was validated. All experts’ recommendations were analyzed considering the adopted theoretical framework, and textual changes and the insertion of information were made for a better understanding of the specifications contained in the scenario.

Additionally, also because of expert recommendation, instruments to measure satisfaction and self-confidence with teaching and simulation design were incorporated, as well as the distribution of explanatory pamphlets (developed by the MH) to CHWs at the end of the participant preparation activity.

## DISCUSSION

This is the first Brazilian study to construct and validate a clinical scenario for CHWs, presenting the development and validation of a clinical scenario aimed at teaching CHWs about actively searching for leprosy cases. Thus, it contributes to the scientific advancement in the field of health and nursing, as the literature points to the incipience of simulation scenarios that contextualize community work^(20)^ and the need to expand the applicability of this resource to improve the quality of training and professional qualification for work in PHC services^(21, 22)^. Especially regarding leprosy, research conducted in Brazil highlights the need for training activities for CHWs to carry out leprosy control actions (LCAs) in the territory covered by PHC services^(3, 5, 6, 23)^. In this context, the presence of trained CHWs is associated with higher scores in essential and derivative attributes of PHC in performing LCAs^(5)^.

This study also aligns with a trend observed in the literature, emphasizing the use of established theoretical frameworks^(13, 16)^ in planning clinical simulations and structuring simulated scenarios in various healthcare areas^(9, 10, 14, 22)^. The use of such frameworks creates conditions for the simulation-based learning experience to achieve its objectives and increase participant satisfaction and self-confidence in the pedagogical experience^(10, 24, 25)^.

The validation of these simulated scenarios has also been strongly recommended in the literature^(11, 13)^. To ensure the quality of the analyzed material during the content validation process, certain aspects need to be presented, such as a clear description of the type of validation adopted, primary findings, validity coefficients, and sample calculations (if applicable). This information helps support the credibility of the results and the legitimacy of the study^(26)^. In this regard, it is worth noting that the present study used Pasquali’s 12 criteria^(18)^ to validate the sections of the scenario using the CVI. This index measures the degree to which the content of an instrument accurately reflects what is being measured^(27)^.

In this study, the learning objectives of the clinical scenario align with some key strategic pillars and components of the “Towards Zero Leprosy” global strategy, namely: (i) training in health services to provide quality care; (ii) scaling up leprosy prevention alongside integrated active case detection; and (iii) combating leprosy-related stigma in communities^(1)^. The alignment of these elements indicates that the execution of the clinically validated simulation in this study has the potential to contribute to filling the knowledge gap among CHWs and to enhance their role in the territories concerning health education for the community^(5, 6, 23)^, timely identification of dermatoneurological symptomatic individuals^(6, 28)^, and comprehensive care for people affected by leprosy and their families^(23)^.

The scenario in this study was designed to be executed in the PHC unit or at a location designated for training purposes. Furthermore, its implementation requires but basic materials and a script to prepare the simulated patient. All these elements enable the standardization of team training and the replication of the activity, facilitating its implementation in professional training programs across different services^(29)^.

Utilizing simulated patients in the clinical scenario allows for realistic and authentic representation of situations encountered in the clinical environment, providing participants with genuine and meaningful interactions^(30)^. In this context, it is important to properly train and prepare the simulated patient for the interaction based on the guidelines and specifications contained in the script. As this scenario involves the simulated patient representing a person with a suspected case of leprosy to be identified by the CHW, it is essential that the facilitator, during training, clarifies how the signs and symptoms of the disease typically manifest, considering the information provided in the case script.

In addition to training, the use of *moulage* contributes to the fidelity and realism of the scenario. In the proposed scenario, the simulated patient will wear *moulage* representing reddish skin patches with raised, poorly defined edges, characteristic of the dimorphic clinical form of leprosy, which is prevalent in Brazil at the time of diagnosis^(2)^.

The *moulages* were manufactured with simple, readily available, and low-cost materials such as white glue and basic makeup. The glue simulated the raised edges, while makeup was used to characterize the appearance of the lesion. Using *moulage* in simulated clinical scenarios provides participants with sensory and emotional experiences that closely resemble those they will encounter in their professional practice^(31)^. The experience of the CHW with the *moulage* created for this study may draw their attention to the importance of conducting a more thorough investigation regarding suspected leprosy cases.

Although the clinical scenario developed in this study addresses only the dimorphic clinical form of leprosy, it can be replaced by other forms, and other resources can be used to characterize the simulated patient, such as printing the image of the desired clinical form on a transparent label. Such adjustments would require a review and, if necessary, adaptation of the learning objectives.

It is understood that the proper manufacture and use of *moulage*, combined with the timely training of the simulated patient and the setup of the scenario, are elements of physical fidelity that create favorable conditions for a meaningful learning experience^(31)^. All these attributes contribute to the replication of the validated scenario in various national health contexts as a means of implementing the strategic pillars and key components of the “Towards Zero Leprosy” global strategy^(1)^.

In addition to physical fidelity elements, it is recognized that a successful implementation of clinical simulation to achieve learning objectives and expected outcomes depends on the facilitator’s assertive guidance to ensure psychological fidelity. In this regard, it is essential for the facilitator to have a thorough understanding of the clinical activity and be adequately prepared to conduct it^(32)^.

Regarding the scenario’s checklist, it is important to mention that it includes eight out of the 14 questions from the Leprosy Suspicion Questionnaire (LSQ), an instrument developed for the screening of suspected leprosy cases. The LSQ has demonstrated sensitivity, specificity, and efficiency in detecting cases through active community^(33)^ and prison-based^(34)^ screening activities.

The experts who analyzed the scenario recommended adjustments to improve the material. One was to include the distribution of an educational pamphlet to CHWs at the end of the simulation preparation section as a means to reinforce the information presented in the lecture-dialogue. In this regard, there is evidence that providing participants with information on the central theme of the simulation before its commencement enhances engagement in the activity and optimizes the learning experience^(13, 33, 35)^.

Another recommendation from the experts that was accepted was the incorporation of assessment instruments that allow obtaining information about the participants’ experience with the simulation and the evaluation of the scenario itself. To measure the simulation’s outcomes, the Student Satisfaction and Self-Confidence in Learning^(35)^ questionnaire was selected, as the adopted theoretical framework considers the expected outcomes for the participant to be satisfaction, self-confidence, changes in knowledge, skills, attitudes, and the transfer of learning to the clinical environment^(16)^.

Therefore, the suggestion to use the Simulation Design Scale to assess the outcomes was not accepted, but it will be used to measure other aspects of the simulation, such as objectives and scenario information, facilitator support during the simulated experience, feedback, reflection, and scenario realism^(35)^. The importance of using this instrument in the pilot test is recognized as part of the final validation process^(19)^.

### Study limitations

The study has limitations, primarily regarding data collection, which occurred asynchronously in a virtual environment, preventing the clarification of doubts at the time of filling out the Google Forms® instrument. Nevertheless, this strategy allowed the participation of a greater number of experts, as the analysis and evaluation of the material could be carried out at any time of the day, depending on the availability of these specialists.

Regarding data collection, another limitation was the choice of validation by sections; that is, the set of items was organized into five sections, but the evaluation was done in bulk, not item by item. It is understood that this way of presenting the material for expert analysis favors a more global examination of the contents, making the assessment faster and more practical on one hand, but with the risk of losing important information/recommendations and suggestions on the other. Finally, the pilot test of the clinical scenario with the target audience is yet to be conducted, which is included as the last step in the content validation process^(13)^.

### Contributions to the field

This is a simple scenario, easy to replicate, cost-effective, and flexible to contextual adaptations. Therefore, it is acknowledged that this study could boost the use of clinical simulation as a PHE strategy in the Brazilian Unified Health System. Furthermore, it is believed that making this clinical scenario available will not only train Brazilian CHWs for the timely detection of leprosy but also benefit other global settings where the disease is a public health problem, as well as those with a low leprosy burden.

## CONCLUSIONS

All essential elements for the development of the clinical scenario aimed at teaching CHWs to actively search for leprosy were covered, following the recommended theoretical framework and simulation practices, as well as recommendations from the Brazilian public health authority for the role of these professionals in dealing with the disease.

The validated scenario presented here represents an important tool for PHC since it enables the qualification of CHWs to develop competencies and skills in the active search for dermatoneurological symptomatics. This makes these professionals more critical and reflective in dealing with leprosy in their daily work, where the reality can range from areas with little to high endemicity.

Content validation by nurse specialists in clinical simulation and leprosy made it possible to refine the scenario through adjustments that could otherwise compromise the performance of the simulated activity. Thus, it is concluded that the clinical scenario “Actions for the prevention and control of leprosy: active search for dermatoneurological symptomatics” is suitable for pilot testing according to the target audience and learning objectives.

## Supplementary Material

0034-7167-reben-76-s2-e20230114-suppl01

## Data Availability

https://doi.org/10.48331/scielodata.NGFOXZ

## References

[1] Organização Mundial da Saúde (OMS) (2021). Estratégia Global para a Hanseníase 2021-2030: Rumo a zero hanseníase [Internet].

[2] Ministério da Saúde (BR) (2023). Boletim Epidemiológico Hanseníase 2023.

[3] Rodrigues RN, Arcêncio RA, Lana FCF. (2021). Leprosy epidemiology and the decentralization of control actions in Brazil. Rev Baiana Enferm.

[4] Ministério da Saúde (BR) (2016). Diretrizes para vigilância, atenção e eliminação da hanseníase como problema de saúde pública: manual técnico-operacional [Internet].

[5] Vieira NF, Lanza FM, Martínez-Riera JR, Nolasco A, Lana FCF. (2020). Orientación de la atención primaria en las acciones contra la lepra: factores relacionados con los profesionales. Gac Sanit.

[6] García GSM, Souza EA, Araújo VM, Macedo MSS, Andrade RMA, Ferreira PRS (2022). Territory, neglected diseases and the action of community and endemic combat agentes. Rev Saúde Pública.

[7] Van’t Noordende AT, Lisam S, Ruthindrarti P, Sadiq A, Singh V, Arifin M (2021). Leprosy perceptions and knowledge in endemic districts in India and Indonesia: differences and commonalities. PLoS Negl Trop Dis.

[8] Souza ALA, Feliciano KVO, Mendes MFM. (2015). Family Health Strategy professionals’ view on the effects of Hansen’s disease training. Rev Esc Enferm USP.

[9] Kolb A, Kolb D. (2018). Eight important things to know about The Experiential Learning Cycle. Australian Educational Leader.

[10] Nascimento FC, Araújo APF, Viduedo AFS, Ribeiro LM, Leon CGRMP, Schardosim JM. (2022). Scenario validation for clinical simulation: prenatal nursing consultation for adolescents. Rev Bras Enferm.

[11] Garbuio DC, Oliveira ARS, Kameo SY, Melo ES, Dalri MCB, Carvalho EC. (2016). Clinical simulation in nursing: experience report on the Construction of a scenario. Rev Enferm UFPE.

[12] Lioce L, Lopreiato J, Downing D, Chang T P, Robertson JM, Anderson M (2020). Healthcare Simulation Dictionary.

[13] INACSL Standards Committee (2016). INACSL Standards of Best Practice: SimulationSM Simulation Design. Clin Simul Nursing.

[14] Dias AAL, Souza RS, Eduardo AHA, Felix AMS, Figueiredo RM. (2022). Validation of two clinical scenarios for simulation-based learning for the prevention and control of healthcare-associated infections. Rev Eletr Enferm.

[15] Fehring R. (1987). Methods to validate nursing diagnose. Heart Lung [Internet].

[16] Jeffries P, Adamson K, Rodgers B., Jeffries P. (2015). The NLN Jeffries Simulation Theory.

[17] Souza RS, Oliveira P P, Dias AAL, Simão DAS, Perizali AEB, Figueiredo RM. (2020). Prevention of infections associated with peripheral catheters: construction and validation of clinical scenario. Rev Bras Enferm.

[18] Pasquali L. (2010). Instrumentação psicológica: fundamentos e práticas.

[19] Coluci MZO, Alexandre NMC, Milani D. (2015). Construção de instrumentos de medida na área da saúde. Ciênc Saúde Coletiva.

[20] Herron EK, Nemeth J, Powers KA. (2017). Community health simulation with a standardized patient: exploring the experience. Clin Simul Nursing.

[21] Ramos DF, Matos M P, Viduedo AFS, Ribeiro LM, Leon CGRMP, Schardosim JM. (2022). Nursing consultation in reproductive planning: scenario validation and checklist for debriefing. Acta Paul Enferm.

[22] Pedrollo LFS, Silva AC, Zanetti ACG, Vedana KGG. (2022). Creation and validation of a high-fidelity simulation scenario for suicide postvention. Rev Latino-Am Enfermagem.

[23] Aquino DMC, Monteiro EMLM, Coutinho NPS, Soeiro VMS, Santos TA, Oliveira EM (2023). Círculo de cultura com agentes comunitários de saúde sobre (des)conhecimentos e estigma da hanseníase. Rev Gaúcha Enferm.

[24] Andrade PON, Oliveira SC, Morais SCRV, Guedes TG, Melo GP, Linhares FMP. (2019). Validation of a clinical simulation setting in the management of post partum haemorrhage. Rev Bras Enferm.

[25] Teixeira A, Tavares J P, Cogo ALP. (2022). Satisfaction and self-confidence of nursing students as participants and observers in realistic simulations. Rev Gaúcha Enferm.

[26] Nascimento JSG, Pires FC, Nascimento KG, Regino DSG, Siqueira TV, Dalri MCB. (2021). Methodological quality of validation of studies on simulated scenarios in nursing. Rev Rene.

[27] Souza AC, Alexandre NMC, Guirardello EB. (2017). Psychometric properties in instruments evaluation of reliability and validity. Epidem Serv Saúde.

[28] Fabri RP, Mazzo A, Martins JCA, Fonseca AS, Pedersoli CE, Miranda FBG (2017). Development of a theoretical-practical script for clinical simulation. Rev Esc Enferm USP.

[29] Meska MHG, Mazzo A, Jorge BM, Souza VD, Negri EC, Chayamiti EMPC. (2016). Urinary retention: implications of low-fidelity simulation training on the self-confidence of nurses. Rev Esc Enferm USP.

[30] Presado MHCV, Colaço S, Rafael H, Baixinho CL, Félix I, Saraiva C (2018). Learning with High Fidelity Simulation. Ciênc Saúde Coletiva.

[31] Mazzo A, Miranda FBG, Meska MHG, Bianchini A, Bernardes RM, Pereira JA. (2018). Teaching of pressure injury prevention and treatment using Simulation. Esc Anna Nery.

[32] Bernardes F, Silva CML, Voltan G, Leite MN, Rezende ALRA, Paula NA (2021). Active search strategies, clinicoimmunobiological determinants and training for implementation research confirm hidden endemic leprosy in inner São Paulo, Brazil. PLoS Negl Trop Dis.

[33] Silva CML, Bernardes F, Voltan G, Santana JM, Leite MN, Lima FR (2021). Innovative tracking, active search and follow-up strategies for new leprosy cases in the female prison population. PLoS Negl Trop Dis.

[34] Kaneko RMU, Lopes MHBM. (2019). Realistic health care simulation scenario: what is relevant for its design?. Rev Esc Enferm USP.

[35] Almeida RGS, Mazzo A, Martins JCA, Baptista RCN, Girão FB, Mendes IAC. (2015). Validation to Portuguese of the Scale of Student Satisfaction and Self Confidence in Learning. Rev Latino-Am Enferm.

