# Supplementary material for: Simulation-based training in Leprosy: development and validation of a scenario for community health workers
Source: Rev Bras Enferm. 2023 Dec 8;76(Suppl 2):e20230114. doi: 10.1590/0034-7167-2023-0114 (PMC10704687; doi:10.1590/0034-7167-2023-0114)
Supplement: 0034-7167-reben-76-s2-e20230114-suppl01 [file 0034-7167-reben-76-s2-e20230114-suppl01.pdf]

Título do conjunto de dados: Dados do Manuscript ID REBEn-2023-0114: Treinamento em hanseníase por simulação: construção e validação de cenário à Agentes Comunitários de Saúde

Informações de contato do pesquisador correspondente:
Nome: Fernanda Moura Lanza
Instituição: Universidade Federal de São João del-Rei (UFSJ)
E-mail: fernandalanza@ufsj.edu.br

Data de coleta de dados: a etapa da elaboração do cenário de simulação clínica ocorreu no período de março a agosto de 2021 e a etapa de validação de conteúdo por comitê de especialistas nas áreas temáticas ocorreu nos meses de setembro e outubro de 2021.

Descrição dos dados e arquivo: apresentação do cenário clínico "Ações de prevenção e controle da hanseníase: busca ativa de sintomáticos dermatoneurológicos" que foi validado por especialistas para para o ensino das ações de busca ativa da hanseníase a Agentes Comunitários de Saúde e está apto para o teste piloto.

Descrição dos métodos de coleta dos dados: para a validação de conteúdo por especialistas, o cenário clínico foi subdividido em cinco seções no Google Forms® para avaliação dos 12 critérios de Pasquali mediante escala do tipo Likert com quatro alternativas de resposta.

Descrição dos métodos de processamento dos dados: utilizou-se o Índice de Validade de Conteúdo para determinar o grau de concordância entre os juízes e análise descritiva das recomendações.

CENÁRIO CLÍNICO

**Nome do cenário**: Ações de prevenção e controle da hanseníase: busca ativa de sintomáticos dermatoneurológicos.
**Treinamento**: Qualificação de Agentes Comunitários de Saúde para realização das ações deprevenção e controle da hanseníase (1º encontro: busca ativa de casos).
**Público-alvo**: Agentes Comunitários de Saúde (ACS).
**Local para simulação**: será realizada na própria unidade de Atenção Primária à Saúde (APS) em umconsultório que estiver disponível ou em área destinada para reunião de equipe.<br>**Local para o *debriefing***: poderá ser realizado na mesma área onde foi executada a cena, contanto que os participantes possam sentar-se em círculo com privacidade.
**Propósito da simulação**: ensino.

OBJETIVOS DE APRENDIZAGEM
**Objetivo geral**: capacitar o ACS para realização da busca ativa de sintomáticos dermatoneurológicos no seu território de atuação.
**Objetivos específicos** (comando implícito: espera-se que ao final da simulação o ACS seja capaz de):<br>a) Realizar ações de educação em saúde sobre os sinais e sintomas da hanseníase;<br>b) Eliminar falsos conceitos relativos à hanseníase, valorizando a cultura local como ponto de contato para a construção de novos saberes sobre a doença;<br>c) Identificar queixas dermatoneurológicas durante a visita domiciliar, reconhecendo que podem ser sinais e sintomas da hanseníase;<br>d) Reconhecer a necessidade de encaminhamento de pacientes sintomáticos dermatoneurológicos para avaliação na unidade de APS;<br>e) Realizar o encaminhamento de caso suspeito de hanseníase à unidade de APS para agendamento da avaliação dermatoneurológica.
**RESULTADOS ESPERADOS**<br>Espera-se que a experiência com a simulação clínica contribua para:<br>a) Satisfação, autoconfiança e segurança dos participantes;<br>b) Sensibilização sobre a necessidade de realização da busca ativa de sintomáticos dermatológicos, dando o correto encaminhamento de casos suspeitos para avaliação;<br>c) Fortalecimento das ações de busca ativa da hanseníase na APS.
**Método/estratégia de avaliação**: avaliação formativa − autoavaliação, avaliação por pares e observação direta e *feedback* do facilitador.
**Modalidade**: simulação cênica (participante simulado).
**Preparação dos participantes e recursos didáticos**: será realizada uma sessão de exposição dialogada sobre a atuação do ACS na realização das ações de busca ativa da hanseníase antes da simulação. Essa atividade foi elaborada com base nos seguintes referenciais:<br>- BRASIL. Ministério da Saúde. Secretaria de Vigilância em Saúde. **Diretrizes para vigilância, atenção e eliminação da hanseníase como problema de saúde pública**: manual técnico-operacional. Brasília: Ministério da Saúde, 2016. cap.1, pag. 8. Disponível em: http://portal.saude.pe.gov.br/sites/portal.saude.pe.gov.br/files/diretrizes_para_._eliminacao_hanseniase_-_manual_-_3fev16_isbn_nucom_final_2.pdf<br>- BRASIL. Ministério da Saúde. Secretaria de Vigilância em Saúde. Departamento de Vigilância das Doenças Transmissíveis. **Guia prático sobre a hanseníase**. Brasília: Ministério da Saúde, 2017. p. 55. Disponível em: https://bvsms.saude.gov.br/bvs/publicacoes/guia_pratico_hanseniase.pdf<br>Tempo previsto para a atividade: 10 minutos<br>Os *slides* que serão utilizados estão disponíveis para consulta e *download* em: https://drive.google.com/file/d/1yJw9PbR3orU-JQ2RKOGqXyHF4yrSZI8I/view?usp=share_link<br>Após a sessão de exposição dialogada, será disponibilizado folder desenvolvido pelo Ministério da Saúde, disponível em:

https://drive.google.com/file/d/1MXLlyS9dYhEsZbZe_a-Ze53zAG72vfOm/view?usp=share_link

Tempo previsto para a realização das atividades:

Prebriefing = 5 minutos | *Briefing* = 5 minutos

Simulação =10 minutos | *Debriefing* = 30 minutos

***Prebriefing* (o facilitador deve)**:

a) Identificar as expectativas dos participantes com o ensino baseado na simulação;
b) Informar o objetivo geral dessa simulação;
c) Informar a sequência das sessões (*briefing,* execução do cenário e *debriefing*);
d) Informar a modalidade da simulação – simulação clínica com um participante simulado (paciente visitado);
e) Orientar sobre os papéis do facilitador, dos participantes simulados e dos aprendizes, quais sejam:

- O facilitador guiará as atividades nas sessões (*briefing,* execução do cenário e *debriefing)*;

- O participante simulado será quem receberá a visita em seu domicílio;

- Um participante voluntário vivenciará o cenário, e os demais serão participantes observadores.

f) Estabelecer contrato de ficção: *"Tentem se inserir no contexto de desenvolvimento da atuação profissional, como se vocês estivessem na prática. Este é um ambiente seguro, no qual vocês poderão expor suas opiniões e decisões, mas para isto é necessário que não ocorra a participação externa para que possam maximizar a oportunidade de desenvolvimento de competências profissionais. Entretanto, para que se tenha êxito nesta estratégia de ensino, é importante que compreendam e respeitem os limites da estrutura do local desta atividade."*

g) Conduzir os participantes no reconhecimento do cenário: "*Este cenário representa um domicílio de uma família atendida pela sua equipe da Estratégia Saúde da Família (ESF). É importante que vocêsreconheçam todos os detalhes para que possa facilitar o atendimento. Dessa forma, vocês devem explorar o cenário e os recursos disponíveis nele".*

Briefing: 8:30h. Você, Agente Comunitário de Saúde, foi instruído(a) a realizar, durante as visitas domiciliares, a sensibilização da comunidade sobre a hanseníase, bem como realizar a busca ativa de pessoas com sinais e sintomas sugestivos da doença na sua microárea de atuação. A primeira residência que você visitará será a da família Oliveira (cadastro 48 da sua microárea). Essa família reside na sua microárea há três anos, e no cadastro consta que a família Oliveira é composta por três pessoas: o sr. Sebastião Oliveira, 34 anos, trabalhador informal da construção civil; a sua esposa, a sra. Rosana Oliveira, 36 anos, do lar; e o filho do casal, João Oliveira, 2 anos. Você realizará a visita para esta família.

**Participantes e equipe de simulação**<br>**Participantes**:<br>1 participante voluntário<br>Até 9 participantes observadores<br>**Equipe**:<br>1 facilitador<br>1 participante simulado (Sr. Sebastião Oliveira)
**Materiais, equipamentos e simuladores**<br>**Materiais e equipamentos**:<br>Short, camisa e chinelo para o participante simuladoMesa<br>com cadeiras<br>Adornos para a mesa (exemplo: passadeira ou forro, vaso de flor etc.)<br>Caneta<br>Prancheta com ficha cadastral familiar, individual, ficha visita domiciliar.<br>Álcool em gel para higienização das mãos
**Caracterização e roteiros**<br>**Caracterização do participante simulado**: paciente usará short, camisa e chinelo, tendo uma *moulage* de manchas de hanseníase dimorfa[1] no dorso (é necessário ter mais de seis manchas).<br>A característica das manchas serão: manchas avermelhadas com bordas elevadas mal delimitadas.<br>Material utilizado para *moulage*: cola, sombra ou *blush* com tons mais avermelhados, base da cor da pele ator, papel toalha e pincel de maquiagem.

[1] É a forma mais comum de apresentação da doença (mais de 70% dos casos). Caracteriza-se, geralmente, por mostrar várias manchas de pele avermelhadas ou esbranquiçadas, com bordas elevadas, mal delimitadas na periferia, ou por múltiplas lesões bem delimitadas semelhantes à lesão tuberculoide, porém a borda externa esmaecida (pouco definida). Há perda parcial a total da sensibilidade, com diminuição de funções autonômicas (sudorese e vasorreflexia à histamina) (BRASIL, 2017, p. 12).

Roteiro para participante simulado acompanhante (sr. Sebastião Oliveira):

Família composta por três pessoas: o sr. Sebastião Oliveira de 34 anos; a sua esposa, a sra. Rosana Oliveira, 36 anos; e o filho do casal, João Oliveira, 2 anos. Sebastião e Rosana são casados há nove anos. Moram em uma casa de alvenaria financiada pelo programa Minha Casa, Minha Vida. Imóvel possui dois quartos, sala, cozinha e banheiro. Local bem arejado, embora pequeno. A coleta de lixo acontece a cada dois dias. Residem no bairro Laranjeiras (município de Alegres) há três anos. Todos os três membros da família são cadastrados e acompanhados pela equipe da unidade de APS mais próxima da residência.

Dados pessoais:

Sebastião Oliveira, nascido em 03/04/1987, natural de Santa Quitéria (município localizado a 230 km de Alegres), cartão SUS GFTCBNGHY, sexo masculino, se declara pardo, estudou até a quinta série, é trabalhador da construção civil atualmente na informalidade, recebe auxílio do governo (Bolsa Família). Mudou para a cidade de Alegres aos 18 anos para trabalhar em uma empresa de construção civil na qual permaneceu até o ano passado. Conheceu a sra. Rosana em um evento da igreja. Antes de se casar com ela, Sebastião residia em um alojamento da referida empresa de construção civil e dividia o quarto com mais três pessoas.

Doença atual e preocupações de saúde:

Sebastião Oliveira apresenta as seguintes queixas relacionadas à saúde: (i) manchas avermelhadas no dorso que não coçam e que apareceram há cerca de sete anos; e (ii) câimbras e dormência nas duas pernas, principalmente nos últimos 12 meses. Relata que nunca procurou a unidade de APS para realizar uma avaliação relacionada a essas queixas, uma vez que o serviço funciona apenas nos dias úteis e em horário comercial. Como ele trabalhava com carteira assinada em uma empresa da construção civil, tinha medo de se ausentar do trabalho para realizar um atendimento de saúde, mesmo sabendo que poderia apresentar um atestado de comparecimento. No último ano ficou desempregado, e como está vivendo de trabalhos informais, possui tempo reduzido para cuidar da saúde. Precisa trabalhar o máximo que conseguir para arcar com os compromissos da família.

Hábitos de vida:

Sr. Sebastião nega ser tabagista, faz uso de bebida alcoólica apenas nos finais de semana. Não faz uso de medicação. Não faz atividade física, pois alega não ter tempo. Faz apenas três refeições ao dia (café da manhã, almoço e jantar). Alimentação rica em carboidratos simples. Dorme cerca de oito horas por noite.

Ambiente/espaço físico:

Domicílio da família Oliveira: será necessário montar um ambiente que represente a "copa" de uma residência. A mesa deve ser coberta com uma toalha de mesa ou uma passadeira e dispor de adornos, e as cadeiras devem ser colocadas em volta dela. O participante simulado convidará o(a) ACS a sentar-se à mesa para iniciar a conversa.

**Progressão da cena** (guia a ser seguido pelo facilitador na condução do caso)			
**Tempo**	**Ações esperadas do participante voluntário**	**Falas do participante simulado**	**Pistas/Dicas**
0-2 minutos	- Identificar-se ao participante simulado.<br><br>- Solicitar a identificação da pessoa (participante simulado) que o(a) está recebendo.<br><br>- Perguntar se os outros membros da família Oliveira estão no domicílio.<br><br>- Perguntar se há alguma queixa ou demanda da família Oliveira para a unidade de APS.	Olá bom dia!<br><br>Eu sou Sebastião.<br><br>Rosana não está em casa no momento. Ela e João foram visitar minha sogra.<br><br>Estamos muito bem, não estamos sentindo nada no momento.	- Perguntar quem é a pessoa que bateu na porta da sua casa.<br><br>- Perguntar o nome do(a) ACS.<br><br>- Perguntar ao ACS seé necessário que todos os membros da família estejam no domicíliopara prosseguir a visita domiciliar.<br><br>- Relatar ao ACS que nem ele nem nenhum outro membro da família tem queixa no momento.
2-4 minutos	- Realizar a divulgação dos sinais e sintomas da hanseníase ao sr. Sebastião.<br><br>- Estabelecer um diálogo com o sr. Sebastião a fim de eliminar falsos conceitos relativos à hanseníase, valorizando a cultura local como ponto de contato para construção de novos saberes sobre a doença.<br><br>- Perguntar ao Sr. Sebastião se ele já tomou a vacina BCG ou se ele possui a cicatriz vacinal de BCG no braço direito.	Eu vi uma propaganda na TV sobre essa tal hanseníase mesmo!<br><br>Eu tenho muitos medos e dúvidas em relação à hanseníase. É a antiga lepra, não é?<br><br>Não sei se tomei esta vacina, vou pegar meu cartão para você olhar para mim.	- Vi uma propaganda na TV sobre uma tal de hanseníase. Você poderia me explicar o que é essa doença?<br><br>- Informar ao ACS que você gostaria que ele(a) verificasse o cartão vacinal.

4-6 minutos	- Perguntar se o sr. Sebastião possui manchas esbranquiçadas, acastanhadas ou avermelhadas com perda e/ou ausência de sensibilidade ao calor, à dor e ao tato (queixas dermatológicas[2]).<br><br>- Perguntar se o sr. Sebastião sente formigamentos, choquese câimbras nos braços e pernas, que evoluem para dormência, ou se ele se queima ou se machuca sem perceber (queixas neurológicas[3]).<br><br>- Perguntar há quanto tempo o sr. Sebastião apresenta tais queixas.<br><br>- Identificar se o sr. Sebastião conhece algum parente ou amigo(a) que tratou ou está em tratamento de hanseníase.	Eu tenho algumas manchas avermelhadas nas costas, mas não coçam; elas não me incomodam.<br><br>Eu sinto muita câimbra e dormência nas duas pernas. Estou comendo muita banana, mas não está melhorando.<br><br>Faz sete anos que as manchas apareceram, e a câimbra e dormência começaram há um ano.<br><br>Não conheço ninguém que teve hanseníase. Eu posso ter pegado de alguém?	- Informar ao ACS que você possui manchas avermelhadas nas costas que não coçam e não sabe o que são. Faz muito tempo que elas apareceram, mas não incomodam porque não coçam.<br><br>- Informar ao ACS que tem sentido muitas câimbras e dormência nas duas pernas. Come muita banana, mas a câimbra não passa.<br><br>- Informar ao ACS sobre o tempo de aparecimento dos sinais e sintomas: manchas avermelhadas há aproximadamente sete anos, e câimbras e dormência nas duas pernas principalmente nos últimos 12 meses.<br><br>- Perguntar ao(à) ACS se ele pode ter pegado essas manchas de alguém

[2] Os principais **sinais e sintomas dermatológicos** da hanseníase são (BRASIL, 2017, p. 9):
• Áreas da pele, ou manchas esbranquiçadas (hipocrômicas), acastanhadas ou avermelhadas, com alterações de sensibilidade ao calor e/ou dolorosa, e/ou ao tato;
• Pápulas, tubérculos e nódulos (caroços), normalmente sem sintomas;
• Diminuição ou queda de pelos, localizada ou difusa, especialmente nas sobrancelhas (madarose);
• Pele infiltrada (avermelhada), com diminuição ou ausência de suor no local.

[3] Os principais **sinais e sintomas neurológicos** da hanseníase são (BRASIL, 2017, p.9):
• Formigamentos, choques e câimbras nos braços e pernas, que evoluem para dormência – a pessoa se queima ou se machuca sem perceber;
• Dor, choque e/ou espessamento de nervos periféricos;
• Diminuição e/ou perda de sensibilidade nas áreas dos nervos afetados, principalmente nos olhos, mãos e pés;
• Diminuição e/ou perda de força nos músculos inervados por esses nervos, principalmente nos membros superiores e inferiores e, por vezes, pálpebras.

7-8 minutos	- Perguntar ao sr. Sebastião se ele não importaria em levantar a blusa para ver as manchas que ele relatou.	Posso mostrar sim! Estão nas minhas costas.	
9 minutos	- Perguntar se a esposa e o filho apresentam manchas ou áreas da pele com perda ou ausência de sensibilidade ou dormência em alguma parte do corpo.	A Rosana não reclamou comigo sobre isso.<br>No caso do João, eu nunca prestei atenção se tem alguma mancha.	- Perguntar ao ACS se é importante verificar se sua esposa e seu filho possuem manchas na pele.
10 minutos	- Encaminhar o sr. Sebastião (caso suspeito) para avaliação dermatoneurológica pelo médico e/ou enfermeiro na unidade de APS.	Que bom que você vai me encaminhar para avaliação dessas manchas!<br>Vou aproveitar para olhar o que podem ser a câimbra e a dormência.	- Perguntar ao ACS seele consegue agendar consulta com o médico ou com a enfermeira para ver as manchas nas costas. Afirmar que aproveitará a consulta para mencionar as queixas relacionadas às câimbras e dormência nas duas pernas.
10 minutos	- Reforçar a necessidade de procurar a unidade de saúde do bairro para agendar a avaliação das suas queixas pelo médico e/ou enfermeiro<br>- Disponibilizar ajuda se for necessário	Como eu agendo a consulta?<br>Muito obrigado!<br>Qualquer dúvida eu procuro você!	- Perguntar ao ACS se ele não conseguir agendar consulta com o médico ou com aenfermeira, se ele(a) pode ajudar a fazer essa marcação

***Checklist* das ações esperadas do participante voluntário**

**Sequência de ações**	**Realizado?**	**Observação**
ACS se identificou ao participante simulado	( ) Não ( ) Sim	
ACS perguntou se há alguma queixa ou demanda da família Oliveira para a unidade de APS	( ) Não ( ) Sim	
ACS mencionou o tema da educação em saúde que foi proposto para eles trabalharem durante as visitas domiciliares daquele mês	( ) Não ( ) Sim	
ACS realizou a divulgação dos sinais e sintomas da hanseníase ao sr. Sebastião	( ) Não ( ) Sim	
ACS estabeleceu um diálogo com o sr. Sebastião a fim de eliminar falsos conceitos relativos à hanseníase, valorizando a cultura local como ponto de contato para construção de novos saberes sobre a doença	( ) Não ( ) Sim	
ACS perguntou se o sr. Sebastião apresenta alguma queixa dermatológica	( ) Não ( ) Sim	
ACS perguntou se o sr. Sebastião apresenta alguma queixa neurológica	( ) Não ( ) Sim	
ACS perguntou há quanto tempo o sr. Sebastião apresenta tais queixas	( ) Não ( ) Sim	
ACS perguntou se o sr. Sebastião conhece algum parente ou amigo(a) que tratou ou está em tratamento de hanseníase	( ) Não ( ) Sim	
ACS realizou a inspeção das manchas localizadas no dorso do sr. Sebastião	( ) Não ( ) Sim	
ACS investigou a situação vacinal de BCG do Sr. Sebastião	( ) Não ( ) Sim	
ACS investigou se os outros membros da família Oliveira apresentam sinais e sintomas dermatoneurológicos	( ) Não ( ) Sim	
ACS encaminhou o caso suspeito de hanseníase (sr. Sebastião) para avaliação do médico e/ou enfermeiro na unidade de APS	( ) Não ( ) Sim	
ACS finalizou a visita domiciliar	( ) Não ( ) Sim	

Debriefing[4]

O roteiro para o *debriefing* está pautado em *Promoting Excellence and Reflective Learning in Simulation* (PEARLS) (EPPICH; CHENG, 2015).

Esclarecimento sobre a sessão de *debriefing*: Gastaremos até 30 minutos com o *debriefing*, que consistirá em quatro fases. Primeiro, terei interesse em saber como vocês estão se sentindo em decorrência deste caso; na sequência, pedirei que vocês descrevam o caso.

Após, iremos explorar ações que foram bem desempenhadas, além daquelas que vocês fariam de maneira diferente. Terminaremos resumindo alguns pontos essenciais do aprendizado para serem aplicados na prática clínica.

Primeiro, as questões serão abertas a quem participou do cenário e, na sequência, aos observadores.

- Inicialmente pedirei ao participante voluntário (informe o nome) que diga como está se sentindo em relação ao caso (em seguida questionar os participantes observadores sobre sentimentos em relação ao caso).
- Agora pedirei a você (nome do participante voluntário) que descreva resumidamente o caso. Quais foram as principais situações com que teve que lidar? O que fez para o paciente? Quais foram as atividades desempenhadas? Pontos-chaves que necessitam ser contemplados:
  - ✓ divulgação dos sinais e sintomas da hanseníase (atividade de educação em saúde);
  - ✓ discussão sobre o estigma em hanseníase;
  - ✓ realização da busca ativa de casos suspeitos de hanseníase por meio do levantamento de queixas dermatoneurológicas;
  - ✓ levantamento do tempo de existência das queixas dermatoneurológicas;
  - ✓ realização da inspeção das manchas localizadas na região dorsal do participante simulado;
  - ✓ levantamento de possíveis vínculos epidemiológicos;
  - ✓ levantamento da situação vacinal;
  - ✓ encaminhamento do caso suspeito de hanseníase para avaliação do médico e/ou enfermeiro na unidade de APS.

Vamos analisar as atividades desempenhadas na simulação. Houve aspectos bem gerenciados, e outros que pareciam desafiadores. Gostaria de falar sobre cada um deles. Quais aspectos você (nome do participante voluntário) considera que foram bem realizados e por que? Quais aspectos da sua atuação você (nome do participante voluntário) gostaria de mudar e por quê? (repetir questionamentos aos observadores). Pontos-chaves que necessitam ser contemplados:

- ✓ divulgação dos sinais e sintomas da hanseníase (atividade de educação em saúde);
- ✓ discussão sobre o estigma em hanseníase;

[4] O *debriefing* é uma sessão realizada após o encerramento do cenário na qual participantes e professor recordam os fatos positivos e as áreas de possíveis melhorias ocorridas durante a atividade (LIOCE *et al.*, 2020).

- ✓ realização da busca ativa de casos suspeitos de hanseníase por meio do levantamento de queixas dermatoneurológicas;
- ✓ levantamento do tempo de existência das queixas dermatoneurológicas; realização da inspeção das manchas localizadas na região dorsal do participante simulado;
- ✓ levantamento de possíveis vínculos epidemiológicos;
- ✓ levantamento da situação vacinal;
- ✓ encaminhamento do caso suspeito de hanseníase para avaliação do médico e/ou enfermeiro na unidade de APS.

- *Caso o facilitador tenha observado alguma lacuna de desempenho durante a simulação, deve proceder um* feedback *diretivo:* Notei que você (citar o comportamento), da próxima vez que for fazer (sugerir o comportamento), porque (informar justificativas). Existem pendências que ainda não foram resolvidas?
- Será aplicada a escala de satisfação e autoconfiança na aprendizagem proposta por Almeida *et al*. (2015).
- Gostaria de finalizar o *debriefing* pedindo que cada um de vocês destaque dois pontos que consideram que ajudarão em sua prática profissional e que serão implementados nela.

REFERÊNCIAS:

ALMEIDA, R.G.S.; MAZZO, A.; MARTINS, J.C.A.; BAPTISTA, R.C.N.; GIRÃO, F.B.; MENDES, I.A.C. Validation to Portuguese of the Scale of Student Satisfaction and Self Confidence in Learning. **Revista Latino-America de Enfermagem**, v.23, n.6, p.1007-13. doi: https://doi.org/10.1590/0104-1169.0472.26.

BRASIL. Ministério da Saúde. Secretaria de Vigilância em Saúde. Departamento de Vigilância das Doenças Transmissíveis. **Guia prático sobre a hanseníase**. Brasília: Ministério da Saúde, 2017. p. 55. Disponível em: https://bvsms.saude.gov.br/bvs/publicacoes/guia_pratico_hanseniase.pdf

EPPICH, W.; CHENG, A. Promoting Excellence and Reflective Learning in Simulation (PEARLS). **Simulation in Healthcare**: The Journal of the Society for Simulation in Healthcare, v. 10, n. 2, p. 106-115, 2015. doi: 10.1097/SIH.0000000000000072
